# Impact of diabetes mellitus on left ventricular longitudinal function of patients with non-ischemic dilated cardiomyopathy

**DOI:** 10.1186/s12933-020-01063-y

**Published:** 2020-06-13

**Authors:** Hidekazu Tanaka, Kazuhiro Tatsumi, Hiroki Matsuzoe, Kensuke Matsumoto, Ken-ichi Hirata

**Affiliations:** Division of Cardiovascular Medicine, Department of Internal Medicine, Kobe University Graduate School of Medicine, 7-5-2, Kusunoki-cho, Chuo-ku, Kobe, 650-0017 Japan

**Keywords:** Diabetes mellitus, Dilated cardiomyopathy, Echocardiography, Heart failure

## Abstract

**Background:**

Left ventricular (LV) longitudinal dysfunction has been identified in type 2 diabetes mellitus (T2DM) patients with preserved LV ejection fraction (LVEF). However, the impact of T2DM on LV longitudinal function or the association of LV longitudinal function with outcome for dilated cardiomyopathy (DCM) remains unclear.

**Methods:**

We retrospectively studied 206 patients with non-ischemic DCM, mean age of 59 ± 17 years and LVEF of 31 ± 8% (all < 45%). All patients underwent a standard echocardiographic examination, and LV longitudinal function was assessed in terms of global longitudinal strain (GLS). Long-term outcomes were assessed, with a median follow-up period of 6.2 years, as primary endpoints of death from or hospitalization for deteriorating heart failure.

**Results:**

GLS of DCM patients with T2DM (n = 55) was significantly lower than that in DCM patients without T2DM (n = 151) in spite of similar conventional LV function (7.0 ± 2.0% vs. 7.8 ± 2.2%, p = 0.03). Kaplan–Meier curves indicated that long-term outcomes for DCM patients without T2DM were better than for those with T2DM (log-rank p = 0.001). Subdividing the two groups into four with by using the median value of GLS (7.9%) showed long-term outcome was worst for DCM patients with T2DM and low GLS. Cox proportional hazards analyses demonstrated an independent association of T2DM, GLS and left atrial volume index with long-term outcome. Moreover, multiple regression analysis for the association of GLS showed that T2DM was the independent determinant parameter for GLS as well as for LVEF and left atrial volume index.

**Conclusion:**

Management of DCM patients with T2DM may be improved by using GLS guidance.

## Background

Type 2 diabetes mellitus (T2DM) is an independent risk factor for cardiovascular disease and its associated mortality [1]. T2DM also contributes to left ventricular (LV) dysfunction and heart failure (HF) independently of coronary artery disease or hypertension [2]. Moreover, T2DM is associated with myocardial fibrosis or increased collagen content and myocardial stiffness [3], and is known as a significant factor associated with coronary artery disease and the development of HF with preserved ejection fraction (HFpEF) [4]. Furthermore, LV longitudinal dysfunction, as assessed in terms of lower global longitudinal strain (GLS), has been identified even in T2DM patients with preserved LV ejection fraction (LVEF) but without overt coronary artery disease or HF [5–13], and it should be considered the first marker of a preclinical form of DM-related cardiac dysfunction, leading to HFpEF [5, 14]. In addition, GLS is reportedly also a better predictor than all other echocardiographic parameters of all-cause mortality in HF with reduced ejection fraction (HFrEF) [15]. Finally, T2DM is also well known as a major cause of HFrEF without coronary artery diseases such as idiopathic dilated cardiomyopathy (DCM). It has been reported that the prognosis of DCM patients with T2DM was worse than that of those without T2DM [16]. However, the impact of T2DM on LV longitudinal function in DCM patients remains unclear. The aim of this study was thus to investigate the impact of T2DM on LV longitudinal function, and the association of LV longitudinal function with outcome for DCM patients.

## Methods

### Study population

The retrospective study group consisted of 215 patients with non-ischemic DCM between June 2010 and March 2019 admitted to Kobe University Hospital, all of whom were diagnosed with reduced LVEF (< 45%). Patients were excluded from enrolment in this study if they met any of the following criteria: (1) history or suspicion of coronary artery disease; (2) previous history of open-heart surgery and congenital heart disease; (3) undeniable secondary cardiomyopathy; (4) serious renal dysfunction defined as glomerular filtration rate < 30 mL/min/1.73 m^2^; (5) uncontrolled hypertension > 180/100 mmHg; and (6) more than moderate primary valvular heart disease other than functional mitral regurgitation. Reduced LVEF due to ischemic cardiomyopathy and LV myocardial ischemia were excluded on the basis of results obtained with coronary angiography, coronary computed tomography angiography, treadmill exercise or stress myocardial perfusion scintigraphy. None of the patients showed an ischemic response, and coronary angiography showed no coronary artery disease, defined as > 50% stenosis of a major epicardial vessel. Nine patients (4.2%) were excluded from all subsequent analyses because of poor echocardiographic image quality, so that eventually 206 patients with DCM were enrolled in this study (Table 1). Their mean age was 59 ± 17 years, LVEF was 31 ± 8% (all < 45%), and 64 patients (31%) were female. The diagnosis of T2DM was based on the World Health Organization criteria [17]. The concentration of biochemical analyses was measured by routine method. Specifically, HbA1c was measured using ADAMS A1c HA-8181 (ARKRAY, Kyoto, Japan), brain natriuretic peptide was measured using AIA-CL2400 (Tosoh, Tokyo, Japan), and estimated glomerular filtration rate was measured using JCA-BM8040G (JEOL, Tokyo, Japan). This study was approved by the local ethics committee of our institution (No. 180038).

**Table 1:** Baseline Characteristics of DCM patients

	Overall DCM patients (n = 206)	DCM patients with T2DM (n = 55)	DCM patients without T2DM (n = 151)	p value
Clinical data				
Age (years)	59 ± 17	62 ± 16	58 ± 18	0.10
Female, n (%)	64 (31)	15 (28)	49 (33)	0.50
Body surface area (m^2^)	1.6 ± 0.2	1.6 ± 0.2	1.6 ± 0.2	0.82
Systolic blood pressure (mmHg)	109 ± 20	107 ± 18	110 ± 21	0.36
Heart rate (bpm)	68 ± 16	69 ± 16	69 ± 17	0.21
NYHA functional class ≥ III, n (%)	42 (20)	14 (26)	28 (19)	0.33
T2DM, n (%)	55 (27)	55 (100)	0 (0)	–
Hypertension, n (%)	40 (19)	10 (18)	30 (20)	0.84
Dyslipidemia, n (%)	58 (28)	21 (38)	37 (25)	0.06
Electrocardiogram				
Atrial fibrillation, n (%)	28 (14)	8 (15)	20 (13)	1.0
QRS duration (msec)	113 ± 23	116 ± 22	112 ± 23	0.24
Blood examination				
HbA1c (%)	6.0 ± 0.8	6.9 ± 0.9	5.7 ± 0.4	<0.0001
BNP (pg/dL)	121 (112–555)	98 (54–212)	127 (54–240)	0.28
eGFR (mL/min/1.73 m^2^)	62 ± 19	58 ± 17	63 ± 19	0.08
Medical treatment (for DCM), n (%)				
ACEI/ARB	200 (97)	53 (96)	147 (97)	1.0
β-blocker	202 (98)	54 (98)	148 (98)	1.0
MRA	99 (48)	29 (53)	70 (46)	0.43
Loop diuretics	109 (53)	35 (64)	74 (49)	0.08
Medical treatment (for T2DM), n (%)				
Insulin	5 (2)	5 (9)	–	–
DPP-4 inhibitor	33 (16)	33 (60)	–	–
GLP-1RA	3 (1)	3 (6)	–	–
Sulfonylurea	8 (4)	8 (15)	–	–
α-GI	6 (3)	6 (11)	–	–
Thiazolidine	2 (1)	2 (4)	–	–
Metformin	33 (16)	33 (60)	–	–
SGLT2 inhibitor	8 (4)	8 (15)	–	–
Echocardiography				
LV end-diastolic volume (mL)	171 ± 56	173 ± 56	170 ± 56	0.79
LV end-systolic volume (mL)	120 ± 48	123 ± 47	119 ± 49	0.56
LV ejection fraction (%)	31 ± 8	30 ± 8	31 ± 8	0.19
Left atrial volume index (mL/m^2^)	51 ± 20	51 ± 25	51 ± 18	0.89
LV mass index (g/m^2^)	128 ± 37	122 ± 32	131 ± 38	0.15
E/e’	14.3 ± 7.9	14.7 ± 7.5	14.1 ± 8.0	0.62
MR ≥ moderate, n (%)	66 (32)	22 (40)	44 (29)	0.13
GLS (%)	7.6 ± 2.0	7.0 ± 2.0	7.8 ± 2.2	0.03

Values are mean ± SD for normally distributed data and median and interquartile range for non-normally distributed data, or n (%)

DCM, dilated cardiomyopathy; T2DM, type 2 diabetes mellitus; NYHA, New York Heart Association; BNP, brain natriuretic peptide; eGFR, estimated glomerular filtration rate; ACEI, angiotensin-converting enzyme inhibitor; ARB, angiotensin II receptor blocker; MRA, mineralocorticoid receptor antagonists; DPP-4, dipeptidyl peptidase-4; GLP-1RA, glucagon like peptide-1receptor agonist; α-GI, α-glucosidase inhibitor; SGLT, sodium glucose cotransporter; LV, left ventricular; E, peak early diastolic mitral flow velocity; e’, spectral pulsed-wave Doppler–derived early diastolic velocity from the septal mitral annulus; MR, mitral regurgitation; GLS, global longitudinal strain

### Echocardiography

All patients underwent a resting standard echocardiographic examination using commercially available echocardiography systems (Aplio Artida: Canon Medical Systems, Tochigi, Japan; Vivid 7 or E9: GE Vingmed Ultrasound AS, Horten, Norway; iE33: Philips Medical Systems, Andover, MA). Digital routine grayscale two-dimensional cine loops from three consecutive heart beats were obtained at end-expiratory apnea from standard parasternal and apical views. Sector width was optimized to allow for complete myocardia visualization while maximizing the frame rate. Standard echocardiographic measurements were obtained in accordance with the current guidelines of the American Society of Echocardiography [18].

### Speckle-tracking strain analysis for GLS

**Fig. 1:**
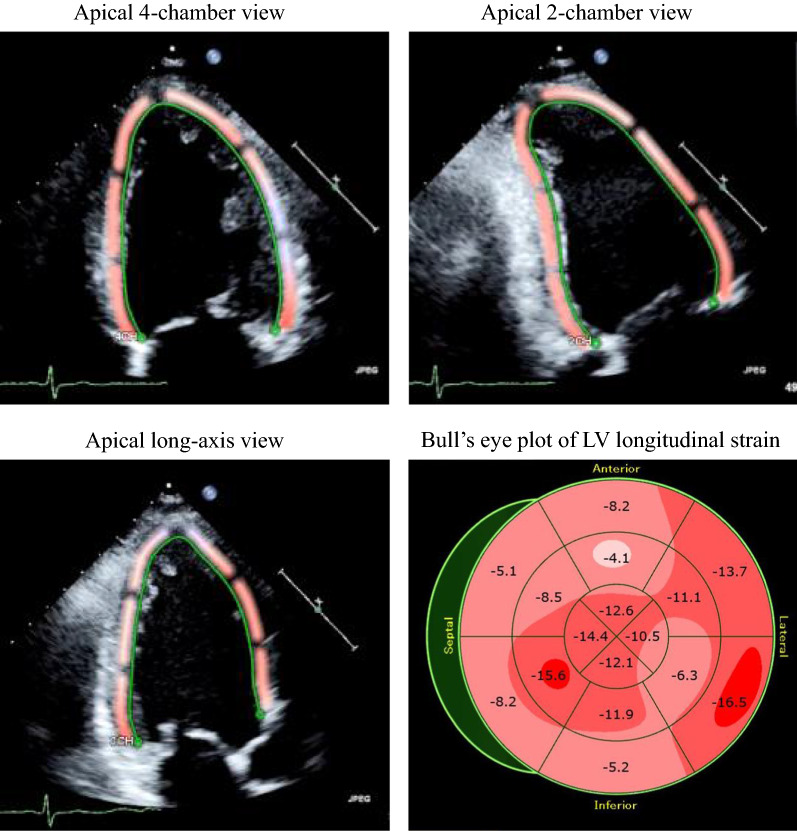
Example of assessment of LV longitudinal myocardial function, known as GLS, by means of two-dimensional speckle-tracking imaging, showing color-coded speckle-tracking images and corresponding bull’s eye plot of LV longitudinal strain

Speckle-tracking strain analysis was performed for each patient with the aid of a single dedicated software to evaluate LV longitudinal function, which was assessed in terms of GLS (AutoSTRAIN, TOMTEC-ARENA: TOMTEC Imaging Systems GmbH, Munich, Germany). Briefly, apical 4-, 2- and long-axis views, obtained as Digital Imaging and Communications in Medicine (DICOM) formatted file images, were uploaded onto a personal computer for subsequent off-line GLS analysis (Fig. 1). Longitudinal speckle-tracking strain was calculated by means of an automated contouring detection algorithm, and manual adjustments of region of interest were performed if necessary. Longitudinal strain results were visualized as color-coded in the individual clips and combined in a bull’s eye plot. GLS was then determined as the averaged peak longitudinal strain of 18 LV segments, and was expressed as an absolute value in accordance with current guidelines [18]. For patients with atrial fibrillation, measurements of GLS was obtained as the averages of ≥ 3 cardiac cycles.

### Definitions of long-term outcome analysis

Long-term unfavorable outcome events were pre-specified as primary endpoints of death from or hospitalization for deteriorating HF over a median follow-up period of 6.2 years (1.9–7.7 years).

### Statistical analysis

Continuous variables were expressed as mean values with standard deviation for normally distributed data and as medians with interquartile range for non-normally distributed data. Categorical variables were expressed as frequencies and percentages. The parameters of the two subgroups were compared by using Student t test or Mann–Whitney U test as appropriate. Proportional differences were evaluated with Fisher’s exact test. Event-free survival curves were determined with the Kaplan–Meier method and cumulative event rates were compared by using the log-rank test. The associations of clinical and echocardiographic parameters with long-term outcomes were identified, by using stepwise selection, with the Cox proportional-hazards model for both univariate and multivariate analyses, and P levels for entry from the model set at < 0.1. Independent associations of GLS with clinical and echocardiographic parameters for DCM patients were evaluated by means of multiple regression analysis. The intraclass correlation coefficient was used to determine inter- and intra-observer reproducibility for GLS from 20 randomly selected subjects. For all steps, a p value of < 0.05 was considered statistically significant. All the analyses were performed with commercially available software (MedCalc software version 18.1.1.; MedCalc Software, Mariakerke, Belgium).

## Results

### Baseline characteristics

The baseline clinical and echocardiographic characteristics of the 206 patients with DCM are summarized in Table 1. T2DM was identified in 55 patients (27%), and the remaining 151 patients (73%) were classified as non-T2DM patients. The intraclass correlation coefficients for inter-observer reproducibility of GLS were 0.979 (95% confidential interval: 0.946–0.989), and the corresponding coefficients for intra-observer reproducibility were 0.970 (95% confidential interval: 0.922–0.986).

### Comparison of GLS of DCM patients with and without T2DM

**Fig. 2:**
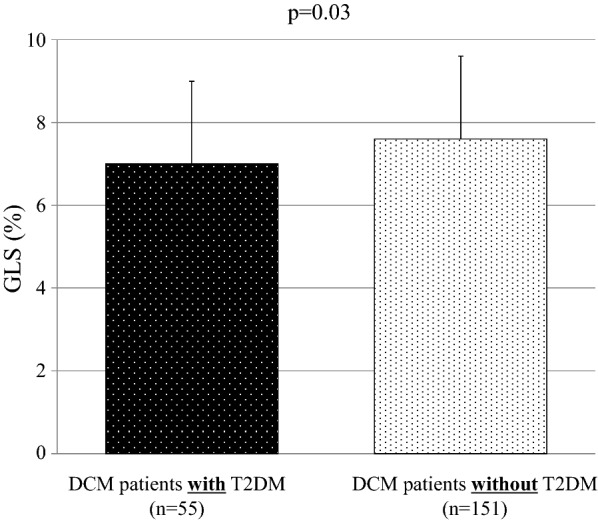
Bar graphs of GLS of DCM patients with and without T2DM showing significantly lower GLS of DCM patients with T2DM despite similar conventional LV function

A comparison of GLS of DCM patients with and without T2DM is summarized in Table 1. Most of the clinical and echocardiographic parameters for the two groups were similar, but GLS of DCM patients with T2DM was significantly lower than of those without T2DM in spite of similar conventional LV function (7.0 ± 2.0% vs. 7.8 ± 2.2%, p = 0.03; Fig. 2).

### Comparison of long-term outcomes for DCM patients with and without T2DM

**Fig. 3:**
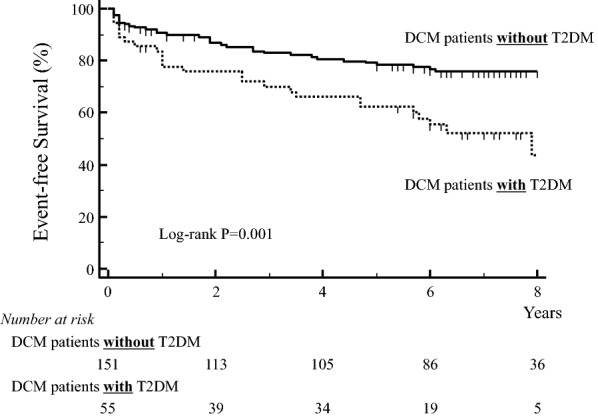
Kaplan-Meier curve shows worse long-term outcome for DCM patients with T2DM than for those without T2DM

The primary endpoint of a pre-specified clinical event occurred in 58 of the 206 patients (28%): 15 deaths from and 43 hospitalizations for deteriorating HF. The Kaplan–Meier curve indicated that long-term outcomes for DCM patients without T2DM were better than for those with T2DM (log-rank p = 0.001; Fig. 3).

### Association of T2DM and GLS with long-term outcome for DCM patients

The hazard ratio (HR) and 95% confidence interval (CI) for each of the variables of the univariate and multivariate Cox proportional hazards analyses are shown in Table 2. An important finding of the multivariate analysis showed that T2DM, GLS and left atrial volume index were independently associated with long-term outcome.

**Table 2:** Univariate and multivariate cox proportional-hazards analysis

Variables	Univariate analysis			Multivariate analysis		
	HR	95% CI	p value	HR	95% CI	p value
Clinical data						
Age	1.00	0.98–.02	0.90			
Female	0.92	0.48–1.77	0.80			
Heart rate	1.00	0.98–1.02	0.72			
NYHA functional class ≥ III	1.86	0.93–3.72	1.86			
T2DM	2.00	1.08–3.69	0.03	1.94	1.11-3.39	0.02
Hypertension	0.82	0.37–1.79	0.61			
Dyslipidemia	0.85	0.44–1.66	0.64			
Electrocardiogram						
Atrial fibrillation	1.74	0.79–3.81	0.17			
QRS duration	1.01	0.99–1.02	0.13			
Blood examination						
BNP	1.00	1.00–1.00	0.84			
eGFR	1.01	0.99–1.03	0.25			
Echocardiography						
LV end-systolic volume	1.00	0.99–1.01	0.99			
LV ejection fraction	1.02	0.96–1.08	0.47			
Left atrial volume index	1.03	1.01–1.05	0.002	1.02	1.01–1.03	0.001
LV mass index	0.99	0.99–1.00	0.24			
E/e’	0.98	0.94–1.02	0.44			
MR ≥ moderate	1.34	0.71–2.55	0.37			
GLS	0.72	0.60–0.86	0.0003	0.75	0.66–0.86	<0.0001

HR, hazard ratio; CI, confidential interval

All other abbreviations as in Table 1

**Fig. 4:**
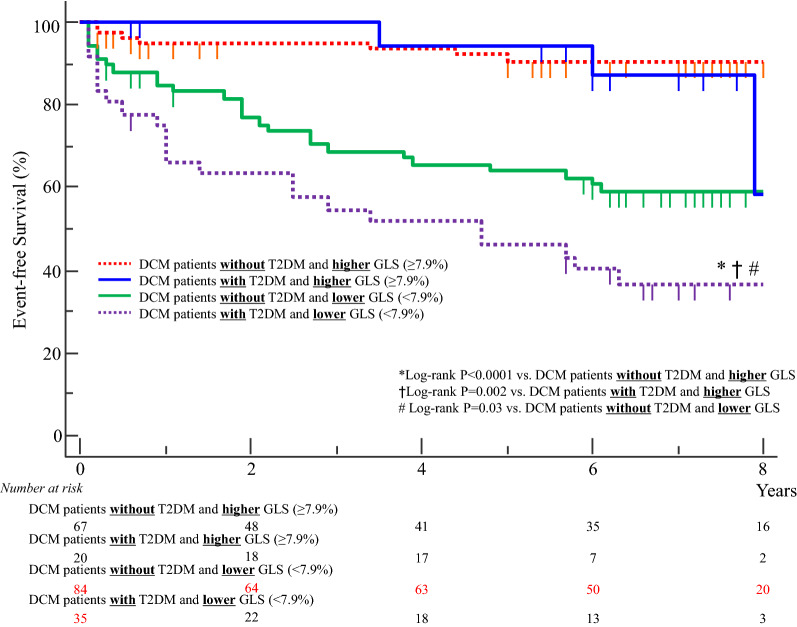
Dividing all 206 DCM patients into two main groups by using the median value of GLS (7.9%) identified 36 DCM patients with T2DM and low GLS. This characteristic was associated with worse long-term outcome compared to the other sub-groups

Next, we divided all 206 DCM patients into two groups by using the median value of GLS (7.9%). There were 36 DCM patients with T2DM and low GLS (< 7.9%). This characteristic was associated with worse long-term outcome than for the other sub-groups (Log-rank p < 0.0001 vs. DCM patients without T2DM and high GLS, Log-rank p = 0.002 vs. DCM patients with T2DM and high GLS, Log-rank p = 0.03 vs. DCM patients without T2DM and low GLS; Fig. 4).

### Association of T2DM with GLS of DCM patients

Table 3 shows the results of multiple regression analysis for the association of GLS with clinical and echocardiographic parameters for DCM patients. An important finding of this analysis was that T2DM proved to be the independent determinant parameter for GLS as well as LVEF and left atrial volume index.

**Table 3:** Multivariate regression analysis for association of GLS

Variables	Coefficient	t value	p value
T2DM	− 0.85	− 2.62	0.01
LV ejection fraction	0.09	3.68	0.0003
Left atrial volume index	− 0.03	− 3.16	0.002

R^2^-adjusted: 0.25

Dependent variables: age, gender (female), heart rate, NYHA functional class ≥ III, T2DM, hypertension, dyslipidemia, atrial fibrillation, QRS duration, BNP, eGFR, LV end-systolic volume, LV ejection fraction, Left atrial volume index, LV mass index, E/e’, MR ≥ moderate

All abbreviations as in Table 1

**Fig. 5:**
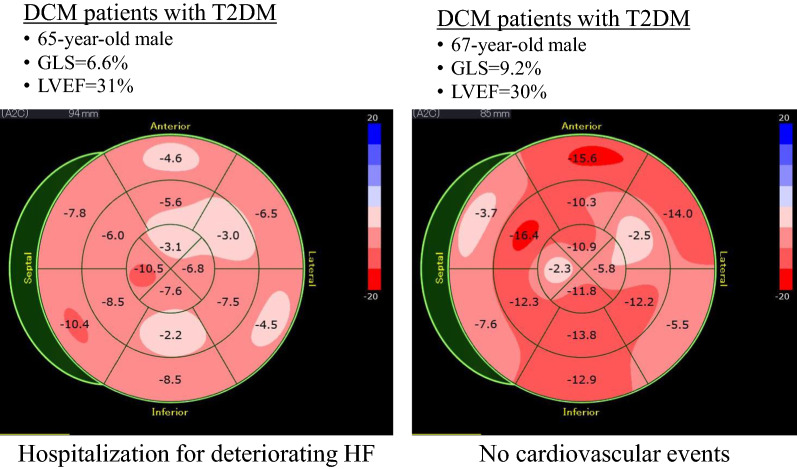
Representative cases of GLS in a bull’s eye plot of DCM patients with T2DM

Figure 5 shows the representative cases of GLS in a bull’s eye plot of DCM patients with T2DM with and without events.

## Discussion

The findings of our study indicate that LV longitudinal function, which was assessed in terms of GLS of DCM patients with T2DM was significantly lower than that of DCM patients without T2DM. In addition, DCM patients with T2DM showed significantly worse long-term outcome than those without T2DM, as did DCM patients with T2DM and reduced GLS. Finally, the presence of T2DM was found to be associated with reduced GLS of DCM patients, and this may be a cause of the worse outcome for DCM patients with T2DM.

### LV longitudinal function in T2DM

T2DM is a well-known risk factor for HF, as well as an important comorbid disease of Stage A HF. Lack of DM control is an important predictor of new onset HF, with every 1% increase in HbA1c correlating to an 8–19% increase in HF incidence [19, 20]. Presence of LV longitudinal dysfunction has been identified in DM patients with preserved LVEF without overt coronary artery disease or HF [5–9, 11, 12, 21–23]. Nakai et al. reported that GLS in T2DM patients was significantly lower than that in age-matched normal subjects in spite of similar LVEF, and that 43% of T2DM patients showed LV longitudinal dysfunction defined as GLS < 17.2% [6], while Ernande et al. found that 23% of T2DM patients with preserved LVEF showed LV longitudinal dysfunction defined as GLS < 18% [8]. T2DM is also a major cause of HFpEF, usually presenting as LV diastolic dysfunction. Some investigators have claimed that LV longitudinal dysfunction, rather than LV diastolic dysfunction, should be considered the first marker of a preclinical form of DM-related cardiac dysfunction in T2DM patients with preserved LVEF and without overt HF [5, 14, 24]. Ernande et al. showed that LV longitudinal dysfunction detected as GLS < 18% was present in T2DM patients with preserved LVEF and even with normal LV diastolic function [5]. This has led to the notion that reduced GLS can coexist with LV diastolic dysfunction, leading to HFpEF, and that GLS can be a more sensitive parameter for predicting subclinical LV dysfunction in T2DM patients with preserved LVEF. It has been also reported that GLS is associated with long-term outcome for Stage A HF patients with T2DM. Holland et al. investigated the association of subclinical LV dysfunction, detected as GLS, with long-term, 10-year outcomes for 230 asymptomatic T2DM patients with preserved LVEF [25]. They found that patients with GLS < 18.9% had significantly worse outcome than those showing a higher percentage, and concluded that GLS was independently associated with the primary endpoint. According to a report by Wang et al. [26], of 290 elderly patients with T2DM and preserved LVEF, those with GLS < 16% showed an increased risk of new-onset of HF and all-cause mortality. Our group also showed that diabetic complications, especially diabetic nephropathy was closely associated with early stage of LV longitudinal myocardial dysfunction in asymptomatic DM patients with preserved LVEF [11]. We showed that hypertriglyceridemia, overweight/obesity, diabetic nephropathy and diabetic neuropathy were independently associated with LV subclinical dysfunction determined as GLS < 18%, with diabetic nephropathy being the highest risk factor in multivariate logistic regression analysis. Furthermore, albuminuria significantly correlated with GLS and a multivariate regression model showed it to be the factor most closely associated with GLS. Also, some investigators reported the association of obesity/overweight with LV function including GLS in T2DM patients [27–30]. Blomstrand et al. showed that overweight and obesity were associated with impaired GLS in both 384 patients with T2DM and 184 patients without T2DM [28].

### T2DM and DCM

T2DM is well known as a major cause of HFpEF or HFrEF with coronary artery disease, but the association of T2DM with HFrEF without coronary artery disease [31], such as seen in DCM, remains uncertain. Previously, LV myocardial hypertrophy and fibrosis have been identified in autopsy studies of T2DM patients who suffered from HF in the absence of atherosclerotic or hypertensive heart disease [32, 33]. Sakakibara et al. reported that their study of 102 consecutive DCM patients showed that the prognosis of DCM patients with T2DM was worse than that of those without T2DM, while multivariate analysis showed that T2DM was significantly associated with an increased incidence of cardiac events [16]. Furthermore, their histological analysis of endomyocardial specimens showed impairment of myocardial relaxation, increased myocardial fibrosis, and mitochondrial degeneration in DCM patients with T2DM, suggesting that this difference between the two groups may be associated with the difference in outcomes. Our study also showed DCM patients with T2DM had worse long-term outcome than those without T2DM, and that reduced GLS in such patients was associated with worse outcome. Furthermore, it has been reported that DCM is characterized by a metabolic shift from fat to carbohydrates and failure to increase myocardial glucose uptake in response to workload increments, and Tricò et al. verified whether this pattern was influenced by an abnormal glucose tolerance [34]. They showed that DCM patients showed a reduced non-esterified fatty acids myocardial uptake, while glucose utilization increased only in DCM patients with abnormal glucose tolerance, but not in DCM patients with normal glucose tolerance. In response to pacing at 130 bpm, glucose uptake promptly rose in non-DCM subject with abnormal glucose tolerance, did not change in DCM patients with abnormal glucose tolerance, and slowly increased in DCM patients with normal glucose tolerance. DCM patients with abnormal glucose tolerance sustained the extra workload by increasing non-esterified fatty acids oxidation, while DCM patients with normal glucose tolerance showed a delayed increase in glucose uptake.

In this study, left atrial volume index was the independent determinant parameter for GLS as well as T2DM and LVEF. It is considered that LV longitudinal myocardial function is closely related to left atrial function (LV size) [10]. Impairment of GLS led to a decrease in the force of drawing the LV basal plane in systole, and this mechanical link to insufficient pulling of the left atrium into the apex. Under such circumstances, poor left atrial compliance because of atrial fibrosis accelerates the reduction of left atrial passive extension, resulting in loss of left atrial reserve. Because the mechanics of left atrial contraction reciprocate against LV pressure as an afterload for left atrial in the pre-atrial contraction period, it plays in the same way an important role for smooth passive stretching in LV end-diastole, whereas a rigid LV characterized as indicating a reduced GLS may lower the left atrial contractile functional evaluation.

### Clinical implications

It has been widely reported that GLS in conjunction with HF stage classification is more useful for HF patient management than conventional echocardiographic parameters, even in patients with findings other than Stage A HF [13]. The utility of GLS for HF patients is accounted for by its ability to predict subclinical LV dysfunction (especially at Stage A), and to identify patients more at risk of progressing to HF stage (especially at Stage B) or to provide details of disease severity or prognosis (especially at Stage C–D). Cameli et al. used assessment of 47 Stage D HF patients by means of Masson’s staining to determine that GLS was strongly associated with LV myocardial fibrosis and its grade [35]. Sengeløv et al. showed that GLS was an independent predictor of all-cause mortality in 1065 HFrEF patients, and GLS was a superior prognosticator compared with all other echocardiographic parameters [36]. Furthermore, Chimura et al. used cardiac magnetic resonance imaging and multivariable analysis of 179 consecutive DCM patients to show that GLS and late gadolinium enhancement were independently associated with long-term outcome [37]. They also found that patients with GLS ≥ 8.3% showed a more favorable long-term outcome than those with lower GLS. Moreover, it has been reported that GLS was found to be useful for predicting fatal ventricular arrhythmias in 94 DCM patients [38]. In our study using DCM patients, T2DM was shown to be associated with a reduction in GLS, which led to worse long-term outcome. Since T2DM is an independent risk factor for cardiovascular disease and its associated mortality, interest in the assessment of risk stratification for DCM patients with T2DM has remained strong. Thus, GLS-guided management using antihyperglycemic drugs as well as cardioprotective drugs for DCM patients with T2DM at a given stage of HF, may be able to prevent progression to later HF stages and offer new insights into the management of DCM patients with T2DM. In fact, prospective studies are currently being conducted to examine the association of antihyperglycemic drugs such as sodium-glucose cotransporter 2 inhibitors or dipeptidyl peptidase-4 inhibitor with GLS in T2DM patients [39–42].

## Study limitations

This study was a single-center retrospective study, so that prospective multi-center studies with larger patient populations will be needed to assess our findings.

## Conclusions

GLS of DCM patients with T2DM was significantly lower than that of DCM patients without T2DM. DCM patients with T2DM showed significantly worse long-term outcome than those without T2DM, as did DCM patients with T2DM and reduced GLS. The presence of T2DM was found to be associated with reduced GLS of DCM patients, and this may be a cause of the worse outcome for DCM patients with T2DM.

## Data Availability

Data sharing not applicable to this article as no datasets were generated or analyzed during the current study.

## Abbreviations

CI: Confidence interval
DCM: Dilated cardiomyopathy
DICOM: Digital imaging and communications in medicine
GLS: Global longitudinal strain
HR: Hazard ratio
HF: Heart failure
HFpEF: Heart failure with preserved ejection fraction
HFrEF: Heart failure with reduced ejection fraction
LV: Left ventricular
LVEF: Left ventricular ejection fraction
T2DM: Type 2 diabetes mellitus
